# The Function and Role of Intercellular Adhesion Molecule 2 in Dental Pulp Cells and Tissue

**DOI:** 10.3390/ijms262412006

**Published:** 2025-12-13

**Authors:** Koudai Tashita, Daigaku Hasegawa, Yuxin Huang, He Zhao, Hidefumi Maeda

**Affiliations:** 1 Department of Endodontology and Operative Dentistry, Faculty of Dental Science, Kyushu University, Fukuoka 812-8582, Japan; tashita@dent.kyushu-u.ac.jp (K.T.); kidsen@dent.kyushu-u.ac.jp (Y.H.); he.zhao@dent.kyushu-u.ac.jp (H.Z.); hide@dent.kyushu-u.ac.jp (H.M.); 2 Division of Endodontology, Kyushu University Hospital, Kyushu University, Fukuoka 812-8582, Japan

**Keywords:** ICAM2, dental pulp cells, odontoblast-like differentiation, direct pulp capping, reparative dentin formation

## Abstract

Direct pulp capping using mineral trioxide aggregate (MTA) is commonly used to preserve dental pulp tissue, but the molecular mechanisms underlying reparative dentin formation during this procedure and the restoration of dental pulp homeostasis remain unclear. To elucidate these mechanisms, we investigated the expression and function of intercellular adhesion molecule 2 (ICAM2) in dental pulp cells and tissue. ICAM2 expression in human dental pulp cells (HDPCs) was confirmed by the gene and protein expression analysis. ICAM2 expression during reparative dentin formation after direct pulp capping was investigated using a rat direct pulp capping model. The effect of ICAM2 on odontoblast-like differentiation of HDPCs was assessed using siRNA and magnetic cell sorting (MACS). The gene and protein expression analysis showed that ICAM2 is expressed in approximately 10% of HDPCs. Immunofluorescence staining of rat mandibular bone sections showed that ICAM2 is expressed in dental pulp tissue. During reparative dentin formation, ICAM2 expression significantly increased to nearly three-fold higher than pretreatment levels on the 3 days after direct pulp capping and then returned to normal levels. ICAM2 knockdown by siRNA enhanced odontoblast-like differentiation of HDPCs. In contrast, culture supernatant from ICAM2-positive HDPCs separated by MACS inhibited odontoblast-like differentiation of HDPCs. These findings suggest that ICAM2 may regulate reparative dentin formation in dental pulp tissue.

## 1. Introduction

Dental caries, traumatic injuries, and dental treatment can cause dentin defects and result in dental pulp exposure. In severe cases of dental pulp exposure or bacterial infection, endodontic treatment is required to remove inflamed and/or infected pulp tissue. Dental pulp, a highly vascularized and innervated tissue located within rigid dentin walls, performs several functions, such as responding to external signals, providing nutrients, and reducing dental sensitivity by repairing pulp through mineralization [1]. After endodontic treatment, teeth are susceptible to altered pulp defense and sensory function due to pulp loss. Moreover, endodontically treated teeth are at greater risk of root fracture than teeth with healthy dental pulp tissue [2,3]. Therefore, preservation of dental pulp tissue can lengthen the lifespan of teeth and thus improve patient health.

Direct pulp capping can maintain pulp vitality in patients with pulp exposure due to caries or trauma. This method involves applying a healing agent directly to exposed pulp, promoting the formation of protective reparative dentin and maintaining pulp vitality [4]. Various materials have been used for direct pulp capping, with the two most common being calcium hydroxide and mineral trioxide aggregate (MTA). These exhibit high biocompatibility, good sealing capacity, antibacterial effects, and promotion of mineralized tissue formation [5,6,7,8]. Recently, MTA has demonstrated a greater ability to maintain pulp tissue integrity than calcium hydroxide. Histological evaluation of exposed pulp tissue-capped with MTA has shown the formation of a thicker dentinal bridge, with low inflammatory response, hyperemia, and pulpal necrosis compared to calcium hydroxide cement [9,10]. Furthermore, MTA appears to induce dentin bridge formation at a faster rate than calcium hydroxide [9,11]. Therefore, it is safe to say that MTA is currently the material of choice for direct pulp capping. However, the molecular mechanisms underlying reparative dentin formation after direct pulp capping with MTA and restoration of dental pulp homeostasis remain unclear. To understand these mechanisms, it is necessary to clarify the molecular signals that promote reparative dentin formation, while also focusing on the anti-mineralization signals that control excessive mineralization of dental pulp tissue.

Intercellular adhesion molecules (ICAMs) are transmembrane glycoproteins belonging to the immunoglobulin superfamily [12]. They play important roles in inflammation, immune responses, and intracellular signaling, and are involved in tissue homeostasis and regeneration [13]. The ICAM family has five members, ICAM1 through ICAM5 [8]. ICAM2, also known as CD102, has been previously well studied and, like the structurally related ICAM1 (CD54) and ICAM3 (CD50), is a known ligand for the ß2 family of leukocyte integrin [14,15,16]. These include CD11a/CD18 (LFA-1) [15,16], CD11b/CD18 (Mac-1) [17], and the dendritic cell-specific, ICAM-grabbing non-integrin (DC-SIGN) [18]. ICAM2 has been reported to play various roles in inflammatory responses and bone metabolism, such as being involved in the proliferation of synovial cells in rheumatoid arthritis [19] and inhibiting bone formation by mediating osteoclast differentiation [20]. However, the role of ICAM2 in mineralization of dental pulp tissue has not been reported. We therefore focused on ICAM2 as a factor that suppresses excessive mineralization of dental pulp tissue during reparative dentin formation after direct pulp capping. ICAM2 may contribute to the restoration of dental pulp homeostasis by suppressing excessive mineralization of dental pulp tissue. In this study, we clarified the expression and localization of ICAM2 in dental pulp cells and tissue and further performed the odontoblast-like differentiation assay using human dental pulp cells (HDPCs) and the rat direct pulp capping experiment. Through these studies, we attempted to demonstrate the function and role of ICAM2 in reparative dentin formation after direct pulp capping.

## 2. Results

### 2.1. ICAM2 Localization in Rat Dental Pulp Tissue and ICAM2 Expression in HDPCs

**Figure 1:**
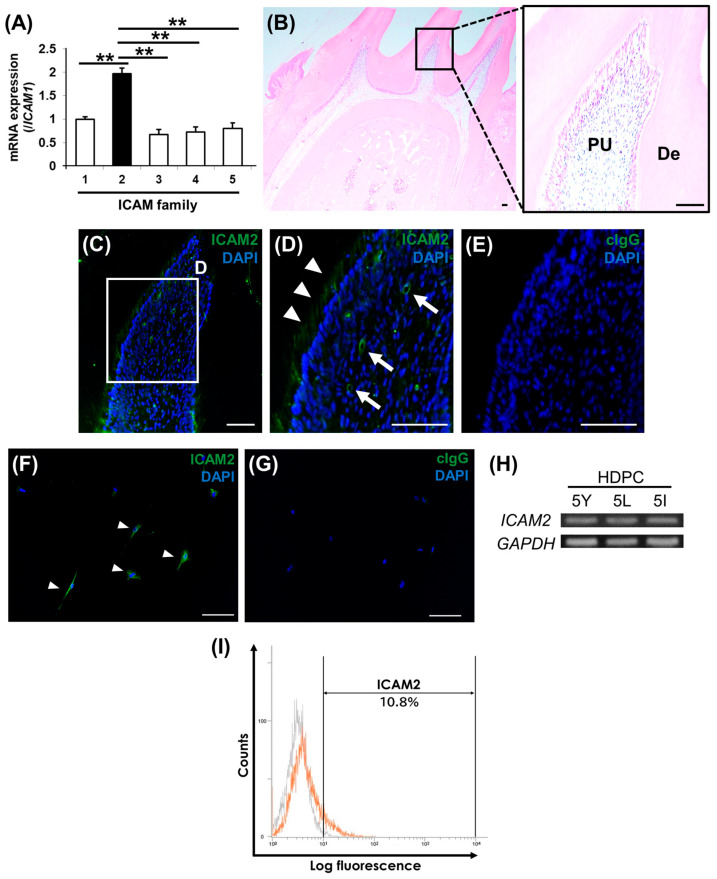
ICAM2 localization in rat dental pulp tissue and ICAM2 expression in HDPCs. (**A**) The mRNA expression of *ICAM1*, *ICAM2* (black column), *ICAM3*, *ICAM4*, *ICAM5* in HDPC-5Y was assessed by quantitative RT-PCR (qRT-PCR). It was normalized against *β-actin* expression (means ± SD; n = 4; ** *p* < 0.01). (**B**) Hematoxylin-eosin (H&E) staining of tissue sections (sagittal sections) of mandibular first molars from Wistar rats. The right panel is the higher magnification view of boxed area in the left panel. PU: dental pulp tissue; De: Dentin. Bars, 100 μm. (**C**–**E**) Immunofluorescence (IF) staining of ICAM2 in the normal dental pulp tissue (**C**). The higher magnification view of boxed area in (**C**,**D**). Positive staining was indicated by arrow heads (odontoblasts) and arrow (dental pulp cells). Negative control: rabbit IgG (cIgG; (**E**)). Nuclei were stained with DAPI (Blue). Bars, 100 μm. (**F**,**G**) The expression of ICAM2 in HDPC-5Y was examined by IF staining. Anti-ICAM2: Green (**F**), anti-rabbit IgG (control IgG: cIgG; (**G**)). Nuclei were stained with DAPI (Blue). Bars, 100 μm. Arrow heads indicate ICAM2-positive HDPCs (**F**). (**H**) The gene expression of *ICAM2* in three HDPCs (HDPC-5Y, 5L, and 5I) was examined by semi-qRT-PCR. It was normalized against *GAPDH* expression. (**I**) The expression intensities of ICAM2 (CD102) of HDPC-5Y (orange line) were demonstrated by flow cytometry. In the gated region, positive cells. Gray line indicates negative control (rabbit IgG).

We first comprehensively analyzed the expression levels of five ICAM family members in human dental pulp cells (HDPCs). Quantitative RT-PCR (qRT-PCR) revealed that *ICAM2* gene was more highly expressed in HDPCs than the other members (Figure 1A). We therefore focused on ICAM2, one of the ICAM members, and aimed to clarify its expression and localization in dental pulp tissue. Hematoxylin-eosin (H&E) staining and immunofluorescence (IF) staining results showed that ICAM2 was expressed in dental pulp tissue, mainly odontoblasts and around blood vessels (Figure 1B–E). We next investigated the expression of ICAM2 in HDPCs in vitro. IF staining showed that ICAM2 was expressed in HDPCs (Figure 1F,G), and semi-qRT-PCR demonstrated that *ICAM2* gene was expressed in HDPCs (Figure 1H). Furthermore, flow cytometry analysis revealed that the percentage of ICAM2-positive HDPCs in the heterogeneous HDPC population was approximately 10% (Figure 1I). These results indicate that ICAM2 is expressed in dental pulp tissue and cells and suggest that ICAM2 may be involved in odontoblast differentiation in dental pulp tissue.

### 2.2. ICAM2 Expression in Rat Dental Pulp Tissue After Direct Pulp Capping

**Figure 2:**
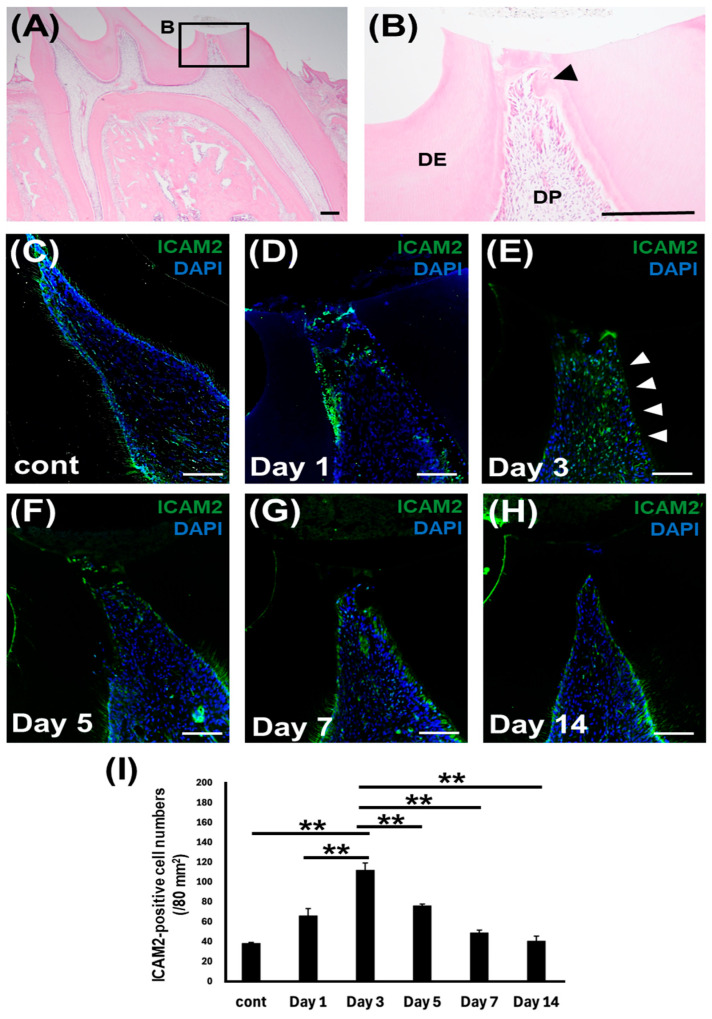
ICAM2 expression in rat dental pulp tissue after direct pulp capping. (**A**,**B**) H&E staining of the rat dental pulp tissue after 7 days of treatment. (**B**) Higher magnification views of the boxed area in (**A**). DP: dental pulp tissue; DE: dentin; Arrowhead: reparative dentin. Bars, 100 μm. (**C**–**I**) IF staining of ICAM2 in the normal dental pulp tissue (**C**) and dental pulp tissue at 1 (**D**), 3 (**E**), 5 (**F**), 7 (**G**), and 14 days (**H**) post-direct pulp capping operation. Anti-ICAM2 (Green); Nuclei were stained with DAPI (Blue). Bars, 100 μm. Arrow heads indicate ICAM2-positive cells (**E**). (**I**) The number of ICAM2-positive DPCs was quantified (means ± SD; n = 3; ** *p* < 0.01).

To further investigate the role of ICAM2 in odontoblast differentiation, we then assessed the dynamics of ICAM2 expression during reparative dentin formation in an established rat direct pulp capping model [21,22]. H&E staining of formalin-fixed, paraffin-embedded tissue showed that 7 days after treatment, induction of reparative dentin had begun, although the exposure site had not yet fully closed (Figure 2A,B). IF staining showed that ICAM2 protein was expressed at low levels in normal rat dental pulp tissue (Figure 2C,I and Figure S1A). One day after direct pulp capping treatment, the number of ICAM2-positive dental pulp cells (DPCs) increased in dental pulp tissue (Figure 2D,I and Figure S1A). Three days after treatment, more intense staining of ICAM2 was detected, even beneath the dental pulp exposure site (Figure 2E,I and Figure S1A). Then, the number of ICAM2-positive DPCs gradually decreased at 5 and 7 days after treatment (Figure 2F,G,I and Figure S1A), reaching the same level as control at 14 days post-surgery (Figure 2H,I and Figure S1A). Staining with a control rabbit IgG was negative in dental pulp tissue (Figure S1B–F). These results demonstrate that during the process of reparative dentin formation, ICAM2 expression significantly increased on the 3 days after direct pulp capping and then returned to normal levels.

### 2.3. Effect of ICAM2 Knockdown on Odontoblast-like Differentiation of HDPCs

**Figure 3:**
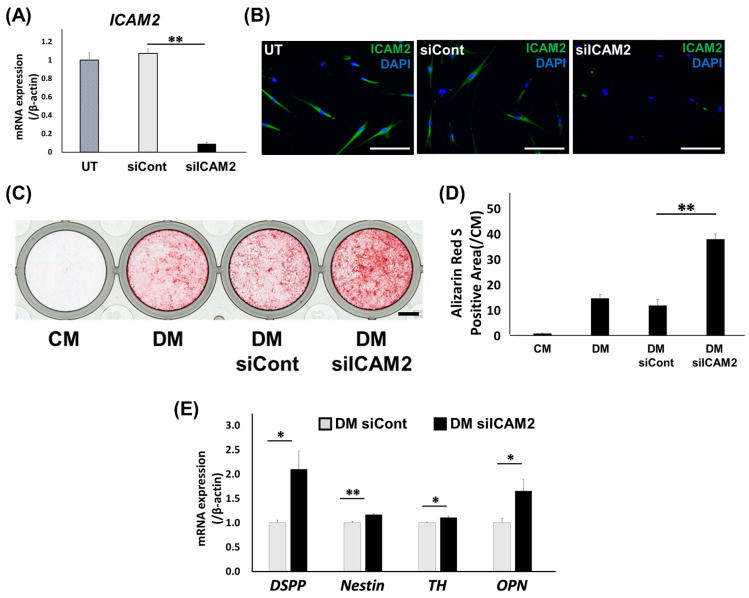
Effect of ICAM2 knockdown on odontoblast-like differentiation of HDPCs. (**A**) The expression of *ICAM2* mRNA in HDPC-5Y transfected with negative control siRNA (siCont.) or ICAM2 siRNA (siICAM2) was assessed by qRT-PCR (UT, Untreated; means ± SD; n = 4; ** *p* < 0.01). (**B**) The expression of ICAM2 in HDPC-5Y was examined by IF staining. Anti-ICAM2: Green; Nuclei were stained with DAPI (Blue). Bars, 100 μm. (**C**,**D**) The formation of mineralized nodules in HDPC-5Y transfected with siCont or siICAM2 was examined by alizarin red S (ARS) staining after culture in odontoblast-like differentiation medium (DM) for 3 weeks. n = 3. The bar, 5 mm. (**D**) The graph shows quantitative analysis of the area of each ARS-positive region, which was imaged and measured using a Biozero digital microscope (means ± SD; n = 3; ** *p* < 0.01). (**E**) The gene expression of *DSPP*, *Nestin*, *TH,* and *OPN* in HDPC-5Y transfected with siCont or siICAM2, which were cultured with DM for 7 days, was assessed by qRT-PCR. It was normalized against *β-actin* expression (means ± SD; n = 4; ** *p* < 0.01, * *p* < 0.05).

We next examined the effect of ICAM2 knockdown on odontoblast-like differentiation of HDPCs. Quantitative RT-PCR and IF staining confirmed that *ICAM2* gene and protein expression in HDPC-5Y was downregulated by 48 h of treatment with ICAM2 siRNA, respectively (Figure 3A,B, Figures S2A and S4A,B). The alizarin red S (ARS)-positive area of HDPC-5Y treated with ICAM2 siRNA (HDPC_siICAM2) cultured in odontoblast-like differentiation medium was larger than that of HDPC-5Y treated with control siRNA (HDPC_siCont) (Figure 3C,D, Figures S2B,C, S3A and S4C–F). In addition, expression of odontoblast-related genes, such as *dentin sialophosphoprotein* (*DSPP*), *Nestin*, *tyrosine hydroxylase* (*TH*) and *osteopontin* (*OPN*), was significantly higher in HDPC_siICAM2 than in HDPC_siCont on day 7 of culture in odontoblast-like differentiation medium (Figure 3E, Figures S2D and S3B). These results suggest that ICAM2 knockdown promotes odontoblast-like differentiation of HDPCs.

### 2.4. Role of ICAM2-Expressing HDPCs on Odontoblast-like Differentiation

**Figure 4:**
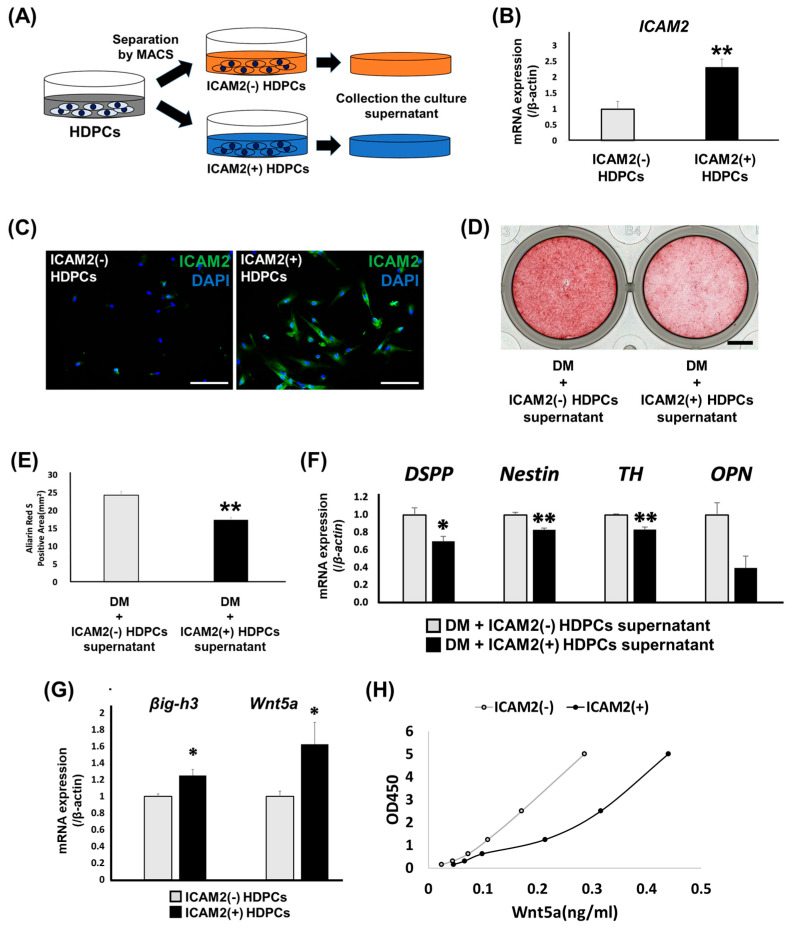
Effect of ICAM2-expressing HDPCs on odontoblast-like differentiation. (**A**) Schema of HDPCs separated using magnetic cell sorting (MACS) and the culture supernatants of each separated cell collected. (**B**) The gene expression of *ICAM2* in ICAM2-negative HDPCs (ICAM2(−) HDPCs) and ICAM2-positive HDPCs (ICAM2(+) HDPCs) after separation using MACS was assessed by quantitative RT-PCR. It was normalized against *β-actin* expression (means ± SD; n = 4; ** *p* < 0.01). (**C**) The expression of ICAM2 in HDPC-5Y was examined by immunofluorescence staining. Anti-ICAM2: Green; Nuclei were stained with DAPI (Blue). Bars, 100 μm. (**D**) The formation of mineralized nodules in HDPC-5Y was examined by ARS staining after culture in DM with ICAM2(−) HDPCs or ICAM2(+) HDPCs supernatant for 3 weeks. n = 3. The bar, 5 mm. (**E**) The graph shows quantitative analysis of the area of each ARS-positive region, which was imaged and measured using a Biozero digital microscope (means ± SD; n = 3; ** *p* < 0.01). (**F**,**G**) The gene expression of odontoblast-related markers (*DSPP*, *Nestin*, *TH* and *OPN*) (**F**) and mineralization-inhibitory factors (*βig-h3* and *Wnt5a*) (**G**) in ICAM2(−) HDPCs and ICAM2(+) HDPCs were assessed by quantitative RT-PCR. It was normalized against *β-actin* expression (means ± SD; n = 4; ** *p* < 0.01, * *p* < 0.05). (**H**) The concentration of secreted Wnt5a protein in ICAM2(−) HDPCs and ICAM2(+) HDPCs supernatant was determined by ELISA.

To further clarify the role of ICAM2 in dental pulp tissue, we isolated ICAM2-positive HDPCs from a heterogeneous HDPC population using the magnetic-activated cell sorting (MACS) and analyzed the effect of these cells on odontoblast-like differentiation. We used MACS to separate HDPCs into ICAM2-positive and ICAM2-negative HDPCs, cultured the cells in normal medium for 48 h and then collected the culture supernatant (Figure 4A). We confirmed ICAM2 expression in the sorted cells using qRT-PCR and IF staining and found that ICAM2-positive HDPCs had significantly higher ICAM2 gene and protein expression than ICAM2-negative HDPCs, respectively (Figure 4B,C and Figure S5A,B). We next cultured HDPCs in odontoblast-like differentiation-inducing medium supplemented with culture supernatant collected from either ICAM2-positive or ICAM2-negative HDPCs (Figure 4A). The ARS-positive area was significantly smaller in HDPCs treated with ICAM2-positive HDPCs supernatant that in those cultured with supernatant from ICAM2-negative HDPCs (Figure 4D,E and Figure S5C–F). In addition, expression levels of odontoblast-related genes, such as *DSPP*, *Nestin*, *TH*, *OPN*, *DLX3*, and *SP7* was significantly lower in HDPCs treated with ICAM2-positive HDPCs culture supernatant than in those treated with ICAM2-negative HDPCs culture supernatant (Figure 4F and Figure S6A). To investigate the reasons for this, we analyzed the expression of mineralization-inhibitory factors in ICAM2-positive and ICAM2-negative HDPCs. ICAM2-positive HDPCs had higher expression of transforming growth factor-β-induced gene product-h3 (*βig-h3*) and *Wnt5a*, both mineralization-inhibitory factors [23,24], than ICAM2-negative HDPCs (Figure 4G). Furthermore, secreted Wnt5a protein was more abundant in the supernatants of ICAM2-positive HDPCs than ICAM2-negative HDPCs (Figure 4H). These results suggest that ICAM2-positive HDPCs may suppress odontoblast-like differentiation through the action of mineralization-inhibitory factors. However, the neutralizing antibody to Wnt5a did not rescue the effect of this culture supernatant (Figure S6B), so the identity of the mineralization-inhibitory factors in this culture supernatant remains unknown.

## 3. Discussion

Dental pulp tissue contains various cells, including stem cells, fibroblasts and odontoblasts, and is responsible for maintaining tooth vitality, and regulating pain transmission and immune responses [25,26]. Dental pulp stem cells (DPSCs) possess typical characteristics of mesenchymal stem cells, demonstrating plastic adherence and clonogenic properties with multilineage differentiation capabilities, including osteoblast differentiation in vitro [27,28]. When odontoblasts are damaged, DPSCs differentiate into odontoblast-like cells in response to inflammatory stimuli and form reparative dentin, preventing further destruction of pulp tissue and maintaining the dentin/pulp complex [4]. During direct pulp capping, the pulp capping material promotes odontoblast-like differentiation of pulp stem cells, thereby ensuring reparative dentin formation and increasing the probability of pulp protection. Although dental pulp tissue contains stem cells capable of forming hard tissue, it remains non-mineralized unless specifically stimulated, and even after dental pulp capping, only a small amount of reparative dentin is formed locally. This suggests that there is an inhibitory mechanism preventing dental pulp cell differentiation into odontoblast-like cells.

**Figure 5:**
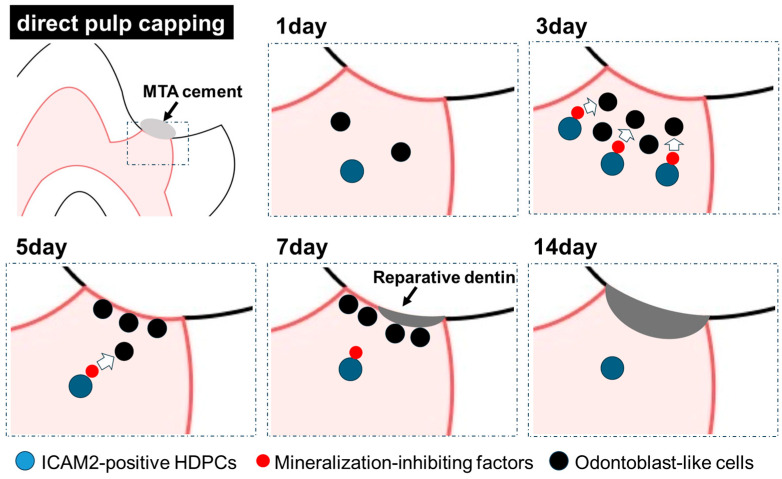
Schematic diagram suggesting the process of reparative dentin formation after direct pulp capping. By the 3 days after direct pulp capping, ICAM2-positive DPCs increased near the pulp capping area, and the mineralization-inhibitory factors secreted by these cells may have suppressed the differentiation of DPCs into odontoblast-like cells. This action may prevent excessive reparative dentin formation after direct pulp capping, restoring dental pulp tissue homeostasis. The dashed box area in the upper left figure indicates the direct pulp capping area (other five figures).

Our recent study showed that reparative dentin formation can be observed 7 days after pulp capping with MTA, with a dentin-bridge covering the exposed pulp area at 14 days post-treatment. These results are consistent with previous studies showing reparative dentin formation 7 days after pulp capping [29,30,31]. MTA releases calcium ions [32,33] and induces reparative dentin when it is applied as a direct pulp capping material on exposed pulp [34,35,36]. During repair of dental pulp tissue after injury, odontoblast-like differentiation of DPCs is essential for synthesis of reparative dentin at the pulp exposure site. Therefore, if reparative dentin is formed 7 days after pulp capping, it is possible that the molecular biological changes leading to odontoblast-like differentiation of pulp cells may be observed 1–5 days after pulp capping. Kuratake et al. reported that cells positive for nestin, a marker for newly formed odontoblast-like cells, appear directly below the pulp capping area on day 3 after direct pulp capping and then arrange themselves at the pulp capping interface on day 5, resulting in the formation of reparative dentin on day 7 [29]. Moreover, previous studies have shown that expression of OPN and DMP1 [30], which regulate odontoblast-like differentiation of pulp cells, is increased near the pulp cap by day 1 after pulp capping. These findings suggest that the molecular signals for odontoblast-like differentiation of dental pulp cells are activated between 1 and 3 days after pulp capping. ICAM2 has been reported to inhibit bone formation by mediating osteoclast differentiation [20]. While the relationship between pulp cell mineralization and ICAM2 has not been previously reported, our animal studies suggest that ICAM2 may play a role in preventing excessive pulp mineralization by inhibiting the promotion of odontoblast-like differentiation in DPCs, thereby maintaining an appropriate level of reparative dentin formation (Figure 5). Our recent studies using a rat model showed that after direct pulp capping with MTA, ICAM2 expression increased immediately beneath the pulp capping site, peaking on day 3. In our pulp capping experiments, ICAM2 expression levels increased sharply on day 3, gradually decreased after day 5, and returned to physiological levels by day 14, which coincides with the formation of a reparative dentin-bridge. These data suggest that once ICAM2 has slowed excessive pulp mineralization, it returns to physiological expression levels (Figure 5). Thus, ICAM2 may play a role in maintaining homeostasis during reparative dentin formation after direct pulp capping.

We performed two in vitro analyses to further clarify the function of ICAM2 after direct pulp capping. An ICAM2 siRNA knockdown assay demonstrated that suppression of ICAM2 in dental pulp cells promotes odontoblast-like differentiation, suggesting that ICAM2 may inhibit odontoblast-like differentiation of dental pulp cells. This indicates that approximately 10% of ICAM2-positive DPCs present in the heterogeneous DPC population suppress odontoblast-like differentiation. ICAM2 is known to have an inhibitory effect on bone metabolism [20]. This finding supports the possibility that ICAM2 also suppresses mineralization in dental pulp tissue. Many reports have been published on factors that promote the differentiation of dental pulp cells into odontoblasts [21,37,38,39,40], but no studies have reported on factors, such as ICAM2 reported in this study, that suppress excessive mineralization of dental pulp tissue and maintain dental pulp homeostasis during reparative dentin formation after direct pulp capping. Excessive mineralization of dental pulp tissue can lead to narrowing of dental pulp tissue even in young adults, which can eventually lead to root fracture. Therefore, we believe that the new mechanism we discovered in this study is novel and will be meaningful in the development of new direct pulp capping materials. In this study, we showed that paracrine application of culture supernatant from ICAM2-expressing human dental pulp cells significantly inhibited odontoblast-like differentiation. These results suggest that ICAM2-expressing dental pulp cells may secrete factors that inhibit odontoblast-like differentiation (Figure 5). Based on previous findings, we speculated that mineralization-inhibitory factors such as βig-h3 and Wnt5a may mediate this process. βig-h3 has been reported to suppress odontoblast-like differentiation of human dental pulp cells [23] and Wnt5a is known to have an inhibitory effect on mineralization in various cells [24,41,42]. Our recent study demonstrated that ICAM2-positive HDPCs expressed significantly higher levels of βig-h3 and Wnt5a than ICAM2-negative HDPCs and further demonstrated that ICAM2-positive HDPCs secreted Wnt5a protein. These findings suggest that mineralization-inhibitory factors such as Wnt5a may be secreted by ICAM2-positive HDPCs and inhibit odontoblast-like differentiation of HDPCs. On the other hand, our recent experiments using neutralizing antibodies have revealed that this mineralization-inhibitory factor is not Wnt5a alone. The next aim of this study is to identify mineralization-inhibitory factors secreted by ICAM2-positive HDPCs, but the results of the Wnt5a-neutralization assay in this study did not lead to its identification. It is difficult to comprehensively identify mineralization-inhibitory factors with our current method, which is the limitation of this study. However, we believe that comprehensive analytical methods such as proteomic analysis may elucidate mineralization-inhibitory factors secreted by ICAM2-positive HDPCs in the future.

## 4. Materials and Methods

### 4.1. Cell Culture

Three HDPC populations were isolated from the healthy third molars of a 29-year-old female (HDPC-5Y), a 21-year-old female (HDPC-5L), and a 24-year-old male (HDPC-5I) after obtaining informed consent from patients who visited Kyushu University Hospital for tooth extraction as described previously [43]. HDPCs were cultured in alpha minimum essential medium (α-MEM; Gibco-BRL, Grand Island, NY, USA) containing 10% fetal bovine serum (FBS; Sigma-Aldrich, St. Louis, MO, USA; 10% FBS/α-MEM) supplemented with 50 µg/mL streptomycin and 50 U/mL penicillin (Gibco-BRL) at 37 °C in a humidified atmosphere of 5% CO_2_ and 95% air. All procedures were approved by the Research Ethics Committee, Kyushu University (approval number: 20A-3).

### 4.2. Quantitative Reverse Transcription Polymerase Chain Reaction (RT-PCR)

Total cellular RNA of HDPCs was isolated with TRIzol Reagent (Invitrogen, Carlsbad, CA, USA), in accordance with the manufacturer’s instructions. The purity and concentration of total RNA were measured using a NanoDrop Lite spectrophotometer (Thermo Fisher Scientific Inc., Walthum, MA, USA). First-strand cDNA was synthesized from 1 μg of total RNA using an ExScript RT Reagent kit (Takara Bio Inc., Kusatsu, Japan). Total RNA was reverse-transcribed with random 6-mers and ExScript RTase for 15 min at 42 °C, and the reaction was stopped by incubation for 2 min at 99 °C, followed by 5 min at 5 °C. PCR was performed using KAPA Express Extract (Kapa Biosystems, Woburn, MA, USA) in a PCR Thermal Cycler Dice (Takara Bio Inc.) under the following conditions: 95 °C for 10 s, then 40 cycles at 95 °C for 5 s and 60 °C for 30 s, followed by a dissociation protocol at 95 °C for 15 s, 60 °C for 30 s, and 95 °C for 15 s. Primer sequences, annealing temperatures, cycle numbers and product sizes for *ICAM1*, *ICAM2*, *ICAM3*, *ICAM4*, *ICAM5*, *DSPP*, *Nestin*, *TH*, *OPN*, *βig-h3*, *Wnt5a* and *β-actin* are shown in Table 1. To calculate the relative mRNA expression, ΔΔCt values were applied using β-actin as an internal calibrator.

### 4.3. Semi-Quantitative RT-PCR

PCR was performed using Platinum Taq DNA polymerase (Invitrogen) in a PCR Thermal Cycler Dice (Takara Bio Inc.) under the following conditions: 94 °C for 2 min and then the appropriate number of cycles at 94 °C for 30 s; appropriate annealing temperature for 30 s; 72 °C for 30 s, and finally 72 °C for 7 min. Primer sequences, annealing temperatures, cycle number, and product sizes for *ICAM2* and glyceraldehyde3-phosphate dehydrogenase (*GAPDH*) are shown in Table 2. GAPDH primers were used as internal standards. All PCR assays were performed within the exponential amplification range. PCR products were separated by electrophoresis on 2% agarose gels (Seakem ME; BioWhittaker Molecular Applications, Rockland, ME, USA) and photographed under ultraviolet excitation after ethidium bromide staining.

### 4.4. Flow Cytometry

The expression of cell surface antigens on HDPCs was analyzed by flow cytometry. Cells (2 × 10^5^/tube) were prepared as a single cell suspension by trypsin/EDTA digestion and resuspended in flow cytometry buffer (R&D Systems, Minneapolis, MN, USA), then incubated with antibodies (10 mg/mL) specific for surface markers or isotype control antibodies (10 mg/mL) on ice for 45 min. Anti-CD102 (ICAM2)-PE antibodies (eBioscience, San Diego, CA, USA) and mouse IgG-PE isotype control were used. Cells were washed with flow cytometry staining buffer and analyzed using an EC800 cell analyzer (Sony Biotechnology, Tokyo, Japan).

### 4.5. Rat Direct Pulp Capping Model

The procedure was conducted as previously reported [21,22]. We used 10-week-old male Wistar rats that were anesthetized with 0.15 mg/kg of medetomidine hydrochloride (Kyoritsu Seiyaku, Tokyo, Japan), 2 mg/kg of midazolam (Sandoz, Tokyo, Japan) and 2.5 mg/kg of butorphanol tartrate (Meiji Seika Pharma, Tokyo, Japan) administered by intraperitoneal injection. The access cavity was created with a #1/2 round steel bur (Dentsply Maillefer, Tienen, Belgium) on the occlusal surface of the upper left first molar and pulp was exposed using a sterile dental explorer. Direct pulp capping was performed with MTA cement (ProRoot, Dentsply Sirona, Charlotte, NC, USA) and covered with glass ionomer cement (Fuji IX, GC Corporation, Tokyo, Japan). The upper right first molar of the same animal served as a control. The animals were transcardially perfused with 4% PFA at 1, 3, 5, 7 and 14 days after treatment. All procedures were approved by the Animal Ethics Committee of Kyushu University (approval number: A20-210-0).

### 4.6. Histological Analysis

Ten-week-old male Wistar rats (Kyudo, Tosu, Japan) with or without direct pulp capping were perfused by intracardiac injection of 4% paraformaldehyde (PFA; Merck Millipore, Darmstadt, Germany) in phosphate-buffered saline (PBS) under anesthesia. The jaws were excised and immersed in 4% PFA for an additional 24 h. The tissue was then washed with PBS and decalcified in 10% ethylenediaminetetraacetic acid (EDTA) for 4 weeks at 4 °C before dehydration and embedding in paraffin. The embedded samples were then sectioned (5 μm in thickness). The sections were observed using a BZ800 microscope (Keyence Corporation, Osaka, Japan) after hematoxylin and eosin (H&E) staining.

### 4.7. Immunofluorescent Staining

Tissue sections were deparaffinized and nonspecific antigens were blocked with 2% bovine serum albumin (BSA; Nacalai Tesque, Kyoto, Japan) in PBS for 1 h at room temperature (RT). A rabbit polyclonal anti-ICAM2 antibody (1:50 dilution; Abcam, Cambridge, MA, USA) and normal rabbit IgG (Cell Signaling Technology, Danvers, MA, USA) were applied as the primary antibody for 1 h at RT. Following washing with PBS, sections were then incubated with an Alexa 488-conjugated chicken anti-rabbit IgG secondary antibody (1:200 dilution; Invitrogen) for 30 min at RT. Subsequently, sections were counterstained with 4,6-diamidino-2-phenylindole (DAPI; Vector Laboratories, Burlingame, CA, USA). Tissue was imaged and analyzed using a BZ-9000 fluorescence microscope (Keyence Corporation). The number of ICAM2-positive cells was counted in an area measuring 400 μm (long) and 200 μm (wide) directly below the pulp capping. Three sections per animal were averaged, and this was performed on three animals (n = 3).

### 4.8. Odontoblast-like Differentiation Assay

HDPCs were seeded at 2 × 10^4^ cells per well in 24-well plates (Becton Dickinson Labware) and cultured in 10% FBS/α-MEM as control medium (CM) or in CM containing 2 mM β-glycerophosphate (Sigma-Aldrich), 50 mg/mL ascorbic acid (Nacalai Tesque) and 10^−7^ M dexamethasone (Merck Millipore) as odontoblast-like differentiation medium (DM) [44]. Half of the medium in each well was exchanged every 2 days. After 4 weeks of culture, cells were fixed with 4% paraformaldehyde (PFA; Merck Millipore) and then washed with distilled water and exposed to alizarin red S (ARS; Sigma-Aldrich) stain as described previously [45]. The ARS-positive regions were imaged and measured using a Biozero digital microscope (Keyence Corporation). Total RNA was isolated from cells after 5 days of culture by a phenol/chloroform method [46] using TRIzol Reagent (Invitrogen) and chloroform (Nacalai Tesque), followed by alcohol precipitation.

### 4.9. Small Interfering RNA Transfection

Small interfering RNA (siRNA) for human ICAM2 (MISSION siRNA, Sigma-Aldrich, St. Louis, MO, USA) or human control siRNA (MISSION siRNA Universal Negative Control; Sigma-Aldrich) were introduced into HDPCs using Lipofectamine RNAiMAX (Invitrogen), according to the method we previously established [24]. Briefly, HDPCs were seeded onto 24-well plates (Becton Dickinson Labware, Lincoln Park, NJ, USA) at a density of 1 × 10^4^ cells per well in Opti-MEM (Invitrogen) containing 10% FBS. After the cells reached 50–70% confluency, siRNA was transduced. The siRNA-lipid complex was prepared by mixing 10 pmol siRNA and 1.5 µL of Lipofectamine RNAiMAX in 50 µL Opti-MEM. The complex was incubated for 5 min at room temperature, then added to cells and the mixture was incubated for 48 h.

### 4.10. Magnetic-Activated Cell Sorting (MACS)

HDPCs were separated into ICAM2-positive and ICAM2-negative HDPCs by MACS using a bead-conjugated antibody against CD102 (ICAM2; Miltenyi Biotec, Bergisch Gladbach, Germany) in accordance with the manufacturer’s instructions. Briefly, 6 × 10^6^ HDPCs were suspended in Flow Cytometry Staining Buffer (R&D Systems, Minneapolis, MN, USA) and centrifuged at 300× *g* for 10 min, resuspended in 80 µL of MACS buffer (Miltenyi Biotec) with 20 µL CD102 microbeads, and incubated in the dark at 4 °C for 15 min. Cells were then washed with MACS buffer, resuspended in 500 µL MACS buffer, and loaded onto an LD column (Miltenyi Biotec) placed in the magnetic field of a MidiMACS separator (Miltenyi Biotec). Unlabeled cells that passed through the column were collected in a tube (ICAM2-negative HDPCs). Labeled cells that remained inside the column were flushed with buffer and collected in a different tube (ICAM2-positive HDPCs).

### 4.11. Enzyme-Linked Immunosorbent Assay (ELISA)

To quantify the concentration of Wnt5a protein secreted from ICAM2-positive HDPCs, a sensitive two-site ELISA was performed. A commercially available ELISA kit (Wnt5a Emax ImmunoAssay System; Promega, Madison, WI, USA) was used to quantify the Wnt5a concentration in culture supernatants of ICAM2-positive HDPCs. The absorbance was measured at 450 nm with Immuno-mini NJ-2300 (Microtec, Urayasu, Japan). All samples and standards were measured in triplicate.

### 4.12. Statistical Analysis

All data were obtained from more than three independent experiments and were presented as mean ± SD. Group comparisons were performed using one-way ANOVA, followed by Tukey’s multiple comparison post hoc test. Student’s unpaired *t* tests were performed for comparisons of two mean values. Statistical significance was defined as a *p*-value < 0.05.

## 5. Conclusions

Our results suggest that ICAM2-expressing pulp cells are recruited to the pulp capping area during reparative dentin formation after pulp capping and secrete proteins that inhibit odontoblast-like differentiation. This may contribute to the restoration of dental pulp homeostasis by balancing the promotion of reparative dentin formation by pulp capping. The findings from this study may help elucidate the poorly understood molecular mechanisms underlying reparative dentin formation and homeostasis after pulp capping and may contribute to the future development of pulp conservation therapy.

## Figures and Tables

**Table 1:** Primer sequences, annealing temperatures, cycle numbers, and product sizes for quantitative RT-PCR.

		Annealing Temperature	Semi-Quantitative RT-PCR	Size of Amplified Products
Gene				
(Abbreviation)	Primer Sequence Forward/Reverse	(°C)	Cycles	(bp)
*ICAM1*	5′-CTTGAGGGCACCTACCTCTG-3′/5′-TTCCGCTGGCGGTTATAGAG-3′	60	40	173
*ICAM2*	5′-CCCTCTTCACCCTGATCTGC-3′/5′-TTCCACTGAGCCTGTTCGTC-3′	60	40	193
*ICAM3*	5′-GGAGATCGTCTGCAACGTGA-3′/5′-CATGCAACTCACGGTCACTG-3′	60	40	142
*ICAM4*	5′-CTCGGCACCCATTACACTGA-3′/5′-CATTTGCATAGGTACGCAGC-3′	60	40	120
*ICAM5*	5′-CCCAGAGAGCTCCGAACCTT-3′/5′-TCGAGGGTGACATCAGGACT-3′	60	40	182
*DSPP*	5′-ATATTGAGGGCTGGAATGGGGA-3′/5′-TTTGTGGCTCCAGCATTGTCA-3′	60	40	136
*Nestin*	5′-TGGCCACGTACAGGACCCTCC-3′/5′-AGATCCAAGACGCCGGCCCT-3′	60	40	143
*TH*	5′-ATGCCGGTACTGGTTCTTCC-3′/5′-TGCCGAAAGGAAATGGGTCA-3′	60	40	90
*OPN*	5′-ACACATATGATGGCCGAGGTGA-3′/5′-TGTGAGGTGATGTCCTCGTCTGT-3′	60	40	115
*βig-h3*	5′-TCCTGAAATACCACATTGGTGATGA-3′/5′-GACATGGACCACGCCATTTG-3′	60	40	160
*Wnt5a*	5′-CTGCAGCCAACTGGCAGGACT-3′/5′-CGCGGCTGCCTATCTGCATCA-3′	60	40	197
*β-actin*	5′-ATTGCCGACAGGATGCAGA-3′/5′-GAGTACTTGCGCTCAGGAGGA-3′	60	40	89

**Table 2:** Primer sequences, annealing temperatures, cycle numbers, and product sizes for semi-quantitative RT-PCR.

		Annealing Temperature	Semi-Quantitative RT-PCR	Size of Amplified Products
Gene				
(Abbreviation)	Primer Sequence Forward/Reverse	(°C)	Cycles	(bp)
*ICAM2*	5′-CCCTCTTCACCCTGATCTGC-3′/5′-TTCCACTGAGCCTGTTCGTC-3′	60	30	193
*GAPDH*	5′-ACCACAGTCCATGCCATCCAC-3′/5′-TCCACCACCCTGTTGCTGTA-3′	60	20	452

## Data Availability

The original contributions presented in this study are included in the article/Supplementary Material. Further inquiries can be directed to the corresponding author.

## Supplementary Materials

The following supporting information can be downloaded at: https://www.mdpi.com/article/10.3390/ijms262412006/s1.

## Abbreviations

The following abbreviations are used in this manuscript:

MTA	mineral trioxide aggregate
ICAM2	intercellular adhesion molecule 2
HDPCs	human dental pulp cells
MACS	magnetic cell sorting
DSPP	dentin sialophosphoprotein
TH	tyrosine hydroxylase
OPN	osteopontin
βig-h3	transforming growth factor-β-induced gene product-h3
DMP1	dentin matrix acidic phosphoprotein 1
RT-PCR	reverse transcription polymerase chain reaction
α-MEM	alpha minimum essential medium
FBS	fetal bovine serum
PFA	paraformaldehyde
PBS	phosphate-buffered saline
EDTA	ethylenediaminetetraacetic acid
H&A	hematoxylin and eosin
GAPDH	glyceraldehyde3-phosphate dehydrogenase
BSA	bovine serum albumin
RT	room temperature
siRNA	small interfering RNA
CM	control medium
DM	differentiation medium
ARS	alizarin red S
