# Supplementary material for: The Function and Role of Intercellular Adhesion Molecule 2 in Dental Pulp Cells and Tissue
Source: Int J Mol Sci. 2025 Dec 13;26(24):12006. doi: 10.3390/ijms262412006 (PMC12732756; doi:10.3390/ijms262412006)
Supplement: Supplementary file 1 [file ijms-26-12006-s001.zip › ijms-3864223-supplementary.pdf]

Supplementary Fig. S1

(A)

	Cont	Day1	Day3	Day5	Day7	Day14
**sample 1**	40	67	101	74	51	33
**sample 2**	38	76	114	76	51	43
**sample 3**	37	55	121	79	45	46

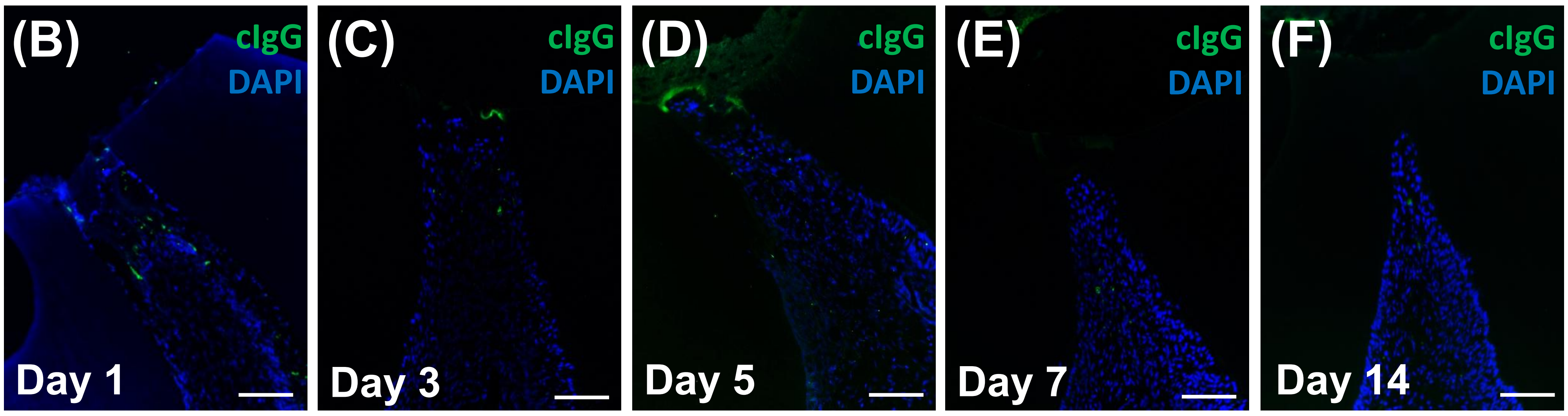

Supplementary Figure S1. Immunofluorescence staining of ICAM2 in the normal dental pulp tissue (Cont) and dental pulp tissue at 1, 3, 5, 7, and 14 days post-direct pulp capping operation. **(A)** Quantitative number of ICAM2-positive cells in each of three samples. **(B)** Staining with a control rabbit IgG (cIgG, Green) in dental pulp tissue. Nuclei were stained with DAPI (Blue). Bars, 100 μm.

Supplementary Fig. S2

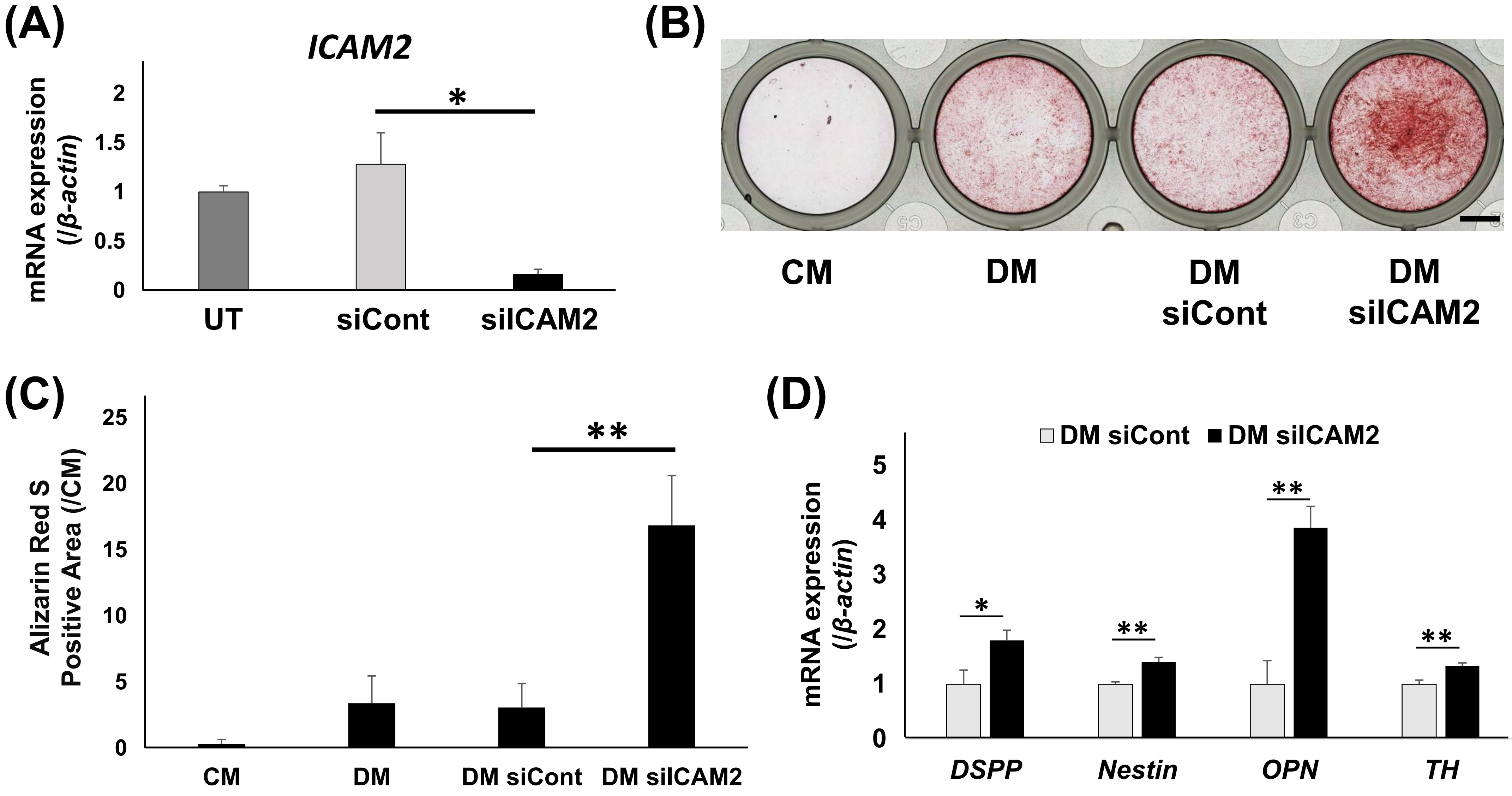

Supplementary Figure S2. Other siRNA knockdown assay data. (A) The expression of *ICAM2* mRNA in HDPC-5Y transfected with negative control siRNA (siCont.) or other ICAM2 siRNA (siICAM2) was assessed by quantitative RT-PCR. (UT, Untreated; means ± SD; n = 4; *p < 0.05) **(B)** The formation of mineralized nodules in HDPC-5Y transfected with siCont or siICAM2 was examined by Alizarin red S (ARS) staining after culture in osteoblastic differentiation medium (DM) for 3 weeks. n = 3. The bar, 5 mm. **(C)** The graph shows quantitative analysis of the area of each ARS-positive region, which was imaged and measured using a Biozero digital microscope. (means ± SD; n = 3; **p < 0.01) **(D)** The gene expression of *DSPP*, *Nestin*, *OPN*, and *TH* in HDPC-5Y transfected with siCont or siICAM2, which were cultured with DM for 7 days, was assessed by quantitative RT-PCR. It was normalized against *β-actin* expression. (means ± SD; n = 4; **p < 0.01, *p < 0.05)

Supplementary Fig. S3

(A)

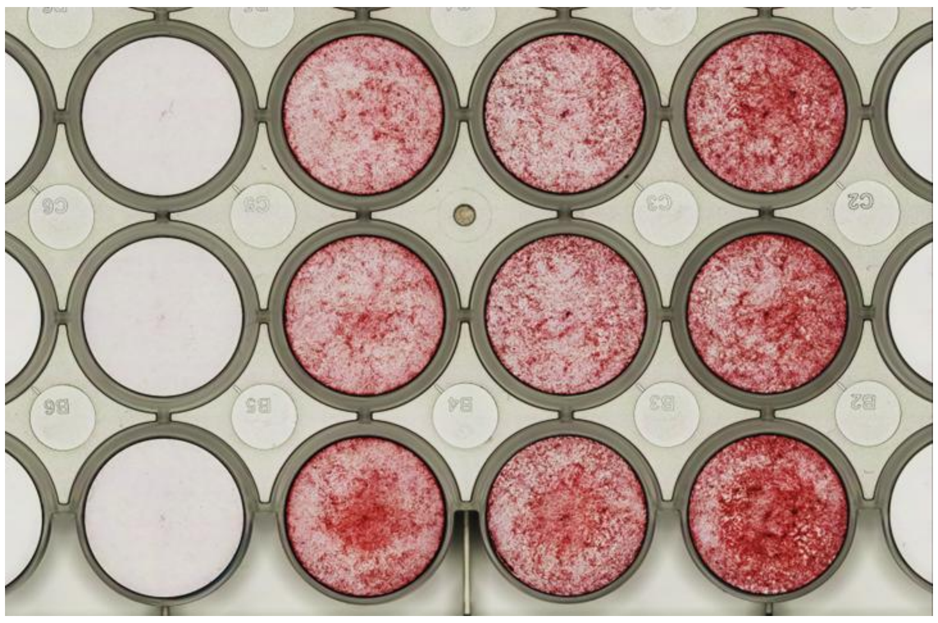

(B)

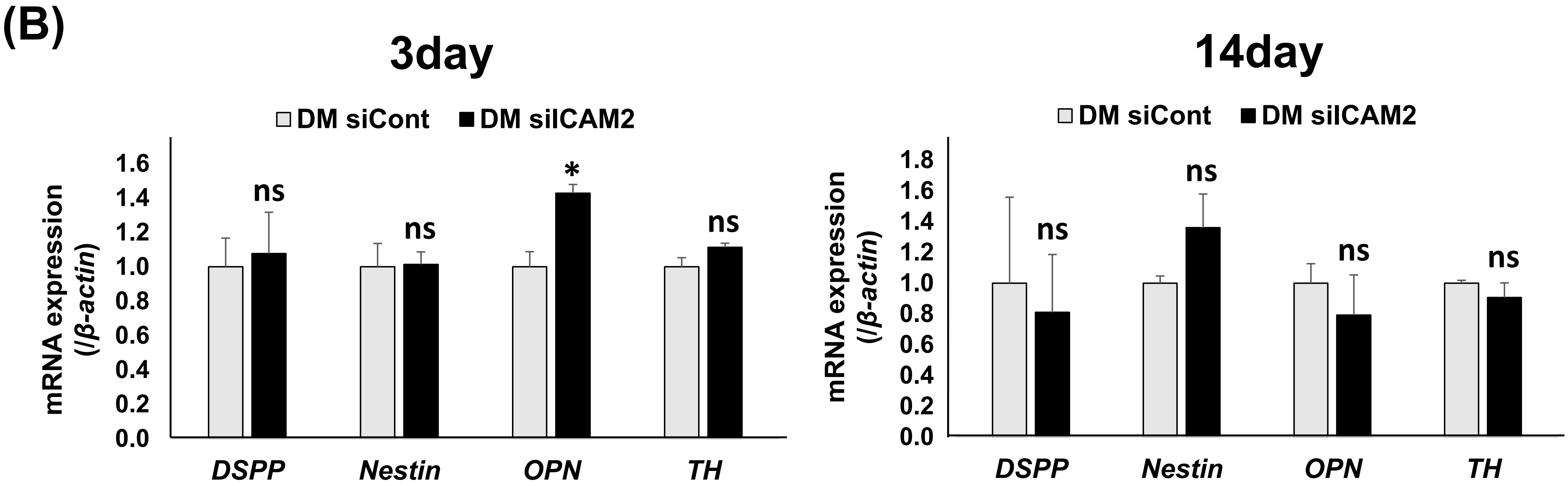

Supplementary Figure S3. (A) The raw image of ARS staining. The formation of mineralized nodules in HDPC-5Y transfected with siCont or siICAM2 was examined by ARS staining after culture in osteoblastic differentiation medium (DM) for 3 weeks. n =3. The bar, 5 mm. **(B)** The gene expression of *DSPP*, *Nestin*, *OPN*, and *TH* in HDPC-5Y transfected with siCont or siICAM2, which were cultured with DM for 3 and 14 days, was assessed by quantitative RT-PCR. It was normalized against *β-actin* expression. (means ± SD; n = 4; *$p < 0.05$, ns: no significant difference)

Supplementary Fig. S4

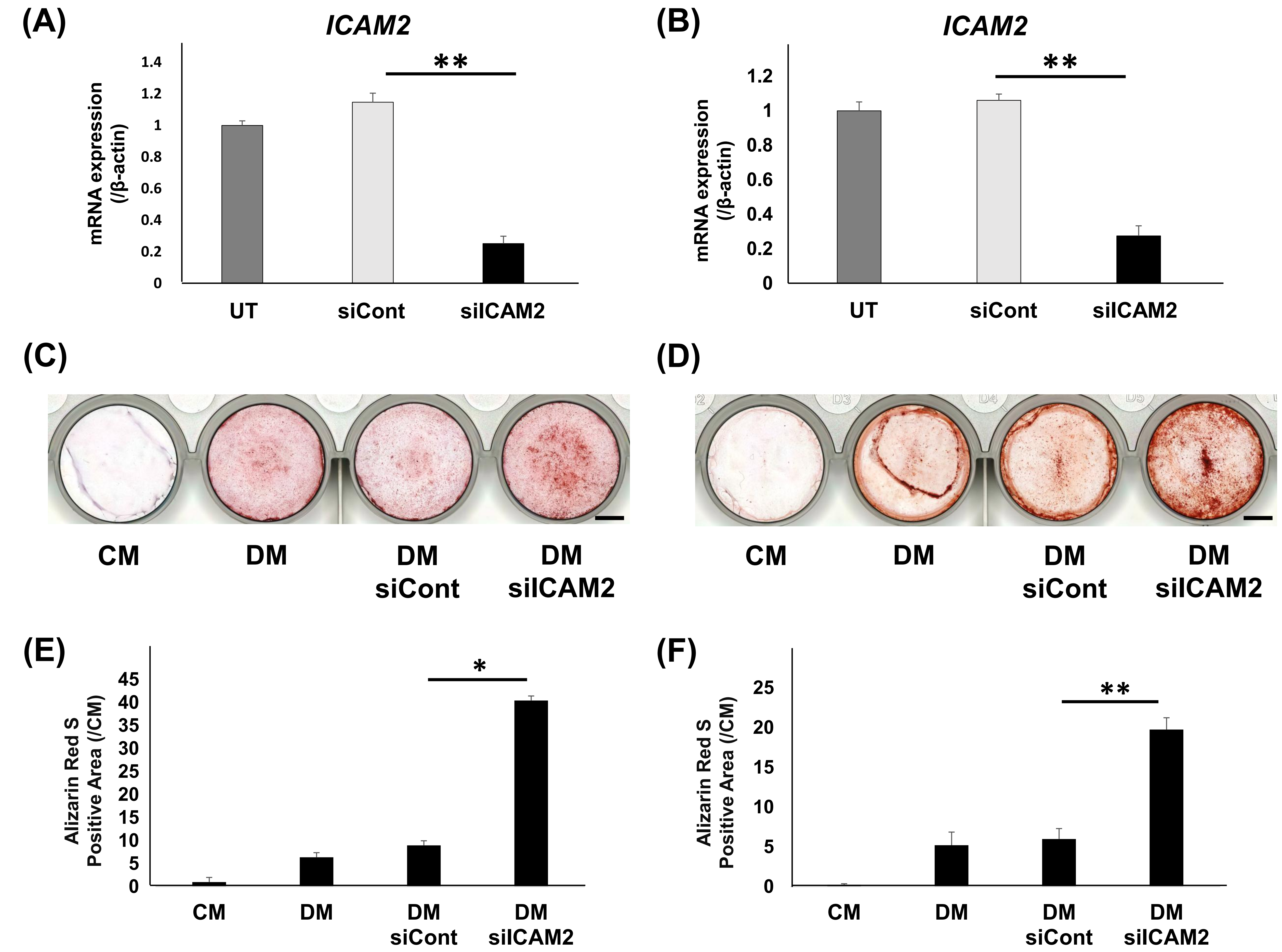

Supplementary Figure S4. Effect of ICAM2 knockdown on odontoblast-like differentiation of HDPCs derived from other donors. **(A, B)** The expression of *ICAM2* mRNA in HDPC-5L **(A)** and HDPC-5I **(B)** transfected with siCont. or siICAM2 was assessed by quantitative RT-PCR. It was normalized against *β-actin* expression. (means ± SD; n = 4; **p < 0.01) **(C, D)** The formation of mineralized nodules in HDPC-5L **(C)** and HDPC-5I **(D)** transfected with siCont or siICAM2 was examined by ARS staining after culture in DM for 3 weeks. Bars, 5 mm. **(E, F)** The graph shows quantitative analysis of the area of each ARS-positive region, which was imaged and measured using a Biozero digital microscope. (HDPC-5L **(E)**, HDPC-5I **(F)**; means ± SD; n = 3; **p < 0.01, *p < 0.05)

Supplementary Fig. S5

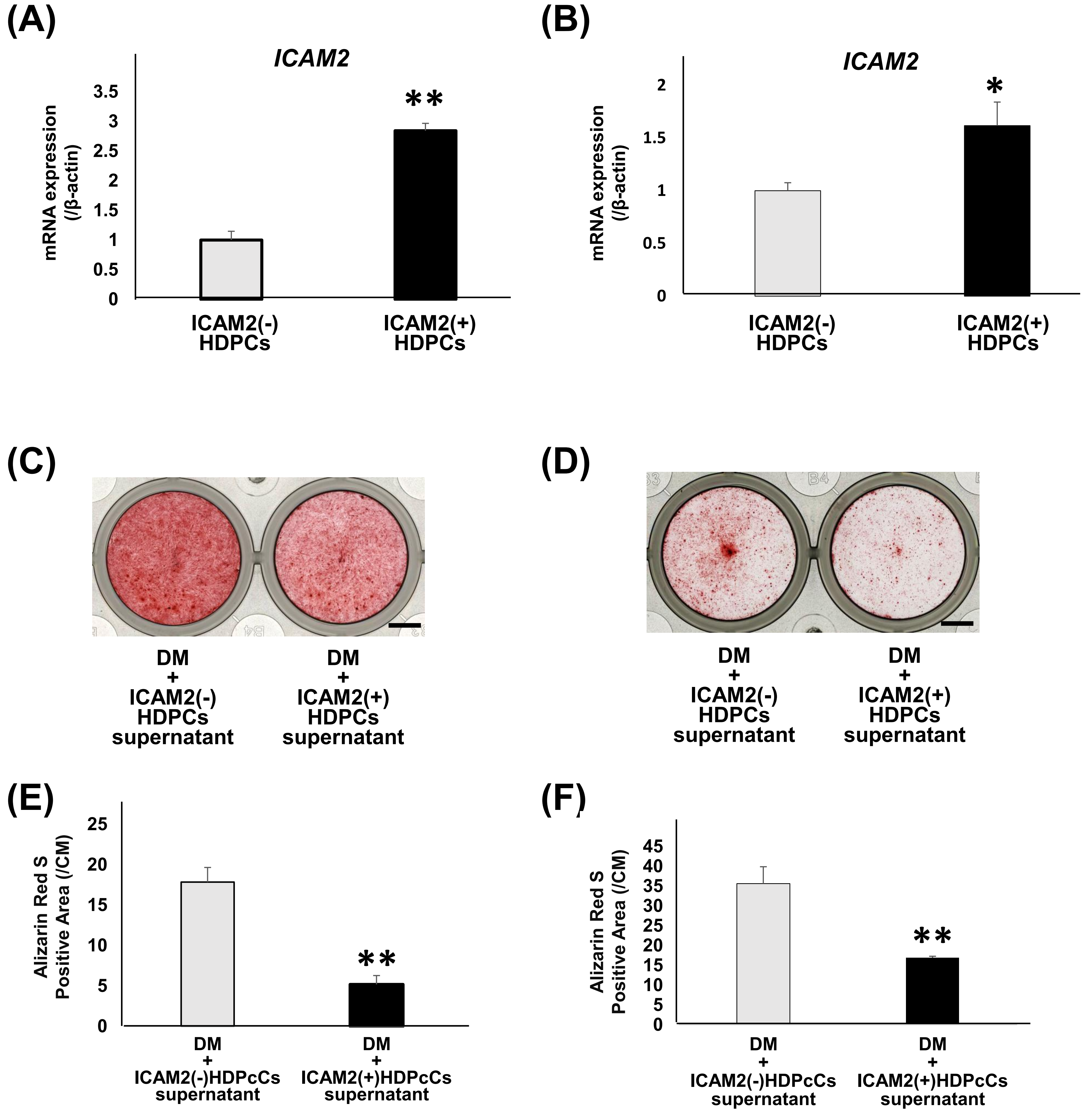

Supplementary Figure S5. Effect of ICAM2-expressing HDPCs culture supernatant on odontoblast-like differentiation in other donors. **(A, B)** The gene expression of *ICAM2* in ICAM2-negative HDPCs (ICAM2(-) HDPCs) and ICAM2-positive HDPCs (ICAM2(+) HDPCs) after separation using MACS was assessed by quantitative RT-PCR. It was normalized against *β-actin* expression. (HDPC-5L **(A)**, HDPC-5I **(B)**; means ± SD; n = 4; **p < 0.01) **(C, D)** The formation of mineralized nodules in HDPCs was examined by ARS staining after culture in DM with ICAM2(-) HDPCs or ICAM2(+) HDPCs supernatant for 3 weeks. (HDPC-5L **(C)**, HDPC-5I **(D)**; n = 3. The bar, 5 mm.) **(E, F)** The graph shows quantitative analysis of the area of each ARS-positive region, which was imaged and measured using a Biozero digital microscope. (HDPC-5L **(E)**, HDPC-5I **(F)**; means ± SD; n = 3; **p < 0.01)

Supplementary Fig. S6

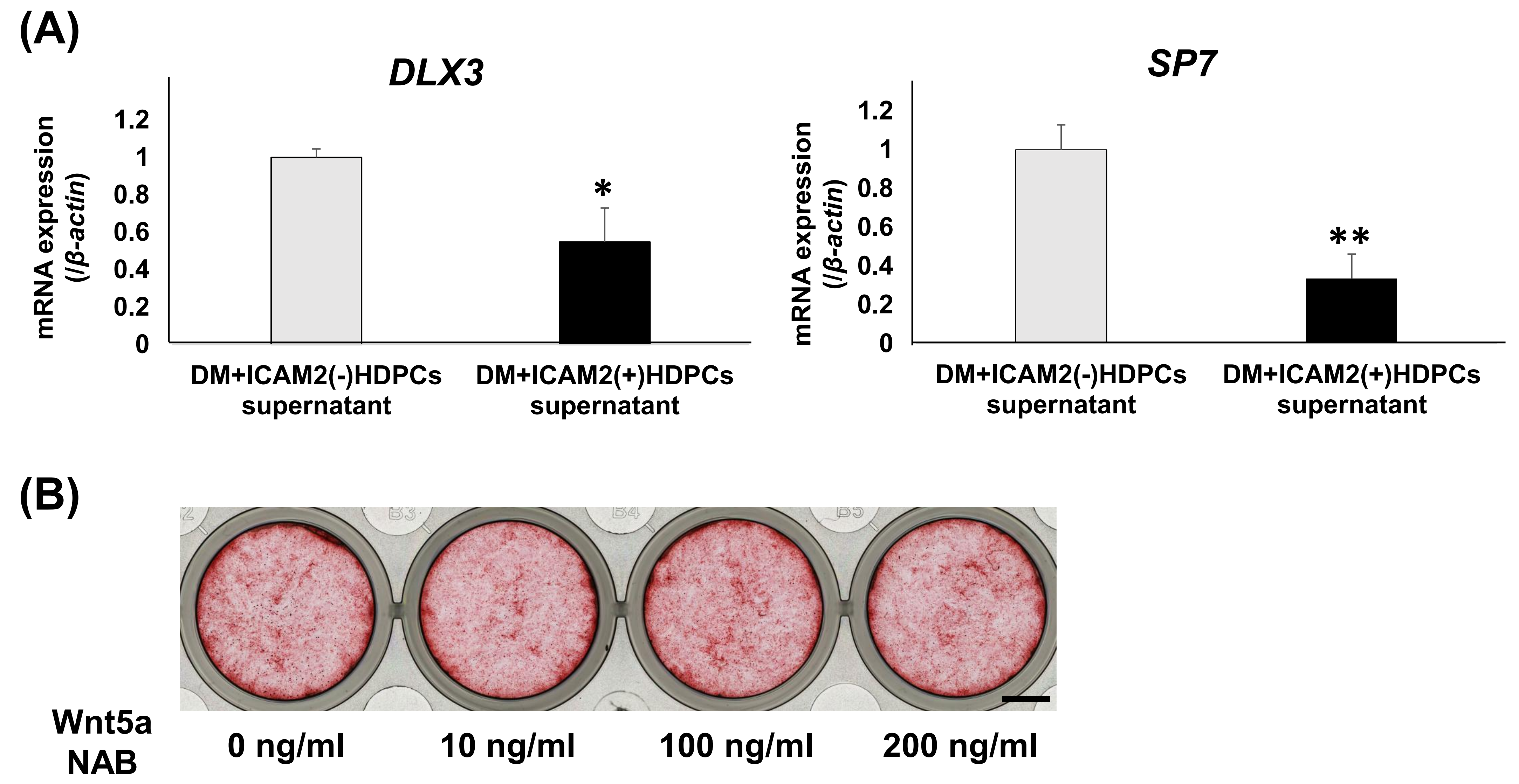

Supplementary Figure S6. (A) The gene expression of *DLX3* and *SP7* in HDPC-5Y cultured in DM with ICAM2(-) HDPCs or ICAM2(+) HDPCs supernatant for 7 days, was assessed by quantitative RT-PCR. It was normalized against *β-actin* expression. (means ± SD; n = 4; $^{**}p < 0.01$, $^{*}p < 0.05$) **(B)** The formation of mineralized nodules after 3 weeks of culturing HDPC-5Y in DM containing ICAM2(+) HDPCs supernatant with 0, 10, 100, and 200 ng/ml Wnt5a-neutralizing was examined by ARS staining. (n = 3. The bar, 5 mm)
